# Efficacy of Health Literacy Interventions for Caregivers of Individuals with Neurodevelopmental and Chronic Conditions: A Rapid Review

**DOI:** 10.3390/children12010009

**Published:** 2024-12-24

**Authors:** Thom Nevill, Jessica Keeley, Susan Hunt, Rachel Skoss, Olivia Lindly, Jenny Downs, Amanda Marie Blackmore

**Affiliations:** 1 The Kids Research Institute Australia, 15 Hospital Ave, Nedlands, WA 6009, Australia; thom.nevill@thekids.org.au (T.N.); jess.keeley@thekids.org.au (J.K.); susan.hunt@thekids.org.au (S.H.); rachel.skoss@nd.edu.au (R.S.); jenny.downs@thekids.org.au (J.D.); 2 Institute of Health Research, The University of Notre Dame Australia, 32 Mouat St., Fremantle, WA 6160, Australia; 3 School of Population and Global Health, The University of Western Australia, 35 Stirling Hwy, Crawley, WA 6009, Australia; 4 Department of Health Sciences, Northern Arizona University, 1100 S Beaver St., Flagstaff, AZ 86011, USA; olivia.lindly@nau.edu; 5 Centre for Child Health Research, The University of Western Australia, 35 Stirling Hwy, Crawley, WA 6009, Australia

**Keywords:** health literacy, health education, caregivers, child, health knowledge, rapid review

## Abstract

Background/Objectives: Caregivers of individuals with neurodevelopmental and chronic health conditions require health literacy (HL) skills for the long-term management of these conditions. The aim of this rapid review was to investigate the efficacy of HL interventions for these caregivers. Methods: Five databases (Cochrane Central, PubMed, Embase, CINAHL, and PsycINFO) were searched. Studies were eligible for inclusion if they reported the efficacy of any intervention aimed at improving the HL of caregivers of individuals with a neurodevelopmental disorder or chronic condition and assessed caregiver HL. All original intervention study designs were eligible, as were systematic reviews. Studies had to be published in English since 2000; grey literature was excluded. The review was registered before commencement with PROSPERO (CRD42023471833). Results: There were 3389 unique records, of which 28 papers (reporting 26 studies) were included. In these studies, 2232 caregivers received interventions through a wide range of media (online, group, written materials, one-to-one, video, phone, and text messages). Research designs were classified as Levels I (*n* = 8), II (*n* = 5), III (*n* = 2), and IV (*n* = 11), and the quality of evidence ranged from high to very low. Half (*n* = 7) of the trials with moderate to high evidence levels reported significant between-group differences in caregiver HL outcomes and/or individuals’ health-related outcomes. Effective interventions occurred across a wide range of conditions, ages, and carer education levels and using a diversity of intervention media. Conclusions: HL interventions for caregivers of individuals with neurodevelopmental and chronic conditions can improve health-related outcomes and caregivers’ HL. Longer and more intensive HL programs may be more likely to be effective, but attention must be paid to participant retention.

## 1. Introduction

Neurodevelopmental conditions are “multifaceted conditions characterized by impairments in cognition, communication, behaviour and/or motor skills resulting from abnormal brain development” [1]. They are usually evident in very early childhood and include global developmental delay, intellectual disability, autism spectrum disorder, attention deficit hyperactivity disorder, as well learning, communication, and motor disorders [2]. A chronic condition is one expected to last at least 6 months, with poor prognosis, having consequences affecting quality of life, with a pattern of recurrence or deterioration [3]. Children with chronic health conditions (e.g., asthma, chronic otitis media, and epilepsy) are developmentally vulnerable in social, emotional, language, cognitive, and physical domains [4].

Children with neurodevelopmental and chronic health conditions are disproportionately high users of healthcare [5,6]. Their conditions usually persist many years and may affect multiple body systems [7]. Their caregivers often have very complex roles, including monitoring adherence to treatments, responding to flare-ups, navigating services, and communicating with healthcare professionals [8,9,10]. These tasks require many skills, including health literacy (HL).

The European Health Literacy Consortium defined HL as entailing “people’s knowledge, motivation and competences to access, understand, appraise, and apply health information in order to make judgments and take decisions in everyday life concerning healthcare, disease prevention and health promotion to maintain or improve quality of life” [11] (p. 3). Responsibility for accessing, understanding, appraising, and applying health information does not rest solely with the individual with a chronic condition but is “distributed” among family members, friends, colleagues, and also community support groups [12]. Therefore, attempts to support these individuals through their healthcare journeys need to target their caregivers as well as themselves. Improving caregiver HL is one way to do this. For the purpose of this review, caregivers are defined as those who provide informal, unpaid support or care in the community (e.g., parents, other family members/relatives, and friends) [13]. Low caregiver HL is sometimes associated with poorer health outcomes, poorer quality of life for the individual and family, and difficulty navigating and communicating with healthcare services [14,15,16,17,18].

The good news is that HL is potentially modifiable: some HL interventions have improved HL, including knowledge and health-related behaviours [16,19]. Based on a systematic review of interventions for caregivers of adults with disabilities and chronic conditions, Yuen et al. suggested tentatively (as evidence is limited) that interventions may be more effective if they tailor information, used mixed modalities, engage a health provider, and use multiple sessions [20]. However, Yuen and colleagues excluded interventions for caregivers of children and adolescents. The present review included interventions for caregivers of individuals of all ages. Its aim was to investigate how HL interventions for caregivers of individuals with neurodevelopmental and chronic conditions affect the caregivers’ HL and the individuals’ health-related outcomes.

## 2. Materials and Methods

### 2.1. Protocol and Registration

This was a rapid review, namely, “a type of knowledge synthesis in which systematic review methods are streamlined and processes are accelerated to complete the review more quickly” [21], following Cochrane Rapid Review Guidelines [21]. Reporting is in accordance with the Preferred Reporting Items for Systematic Reviews [22]. The review was registered before commencement (12 October 2023) with PROSPERO (CRD42023471833) and amended after data extraction (29 February 2024). The amendment did not necessitate another search.

### 2.2. Search Strategy

Cochrane Central, PubMed, Embase, CINAHL, and PsycINFO were searched. Reference lists of selected articles were also scanned. Two authors (T.N. and J.K.) developed search terms, which all authors reviewed. Search terms relating to HL were from prior systematic reviews [23,24,25] and the European Health Literacy Consortium’s definition of HL [11]. A chronic condition was defined as one expected to last at least 6 months, with poor prognosis, having consequences affecting quality of life, with a pattern of recurrence or deterioration [3]. The search terms relating to chronic conditions were from this definition and search terms in prior reviews. The full search terms are in online Supplemental Table S1. The search was conducted 12 October 2023 (Table S1).

### 2.3. Eligibility Criteria

Studies meeting the following criteria were included:

- The sample included caregivers of individuals with neurodevelopmental or chronic conditions.
- The study reported the efficacy of any intervention aimed at improving the HL of these caregivers. HL included accessing, understanding, appraising, and applying health information [11]. Interventions could be delivered in any setting, including clinical, community, educational, or home-based settings or online.
- There was any or no comparison.
- The primary outcome was HL. Secondary outcomes included individuals’ health indicators, healthcare service use, economic costs of healthcare, and health-related adverse events.
- Study designs included systematic reviews, randomized or non-randomized controlled trials, or any other intervention study design. Data could be quantitative, qualitative, or mixed.
- Studies were published in English since 2000, and the full text was available. Grey literature was excluded, including books, dissertations, reports, conference papers, media (e.g., newspaper), letters to editors, editorials, blogs, podcasts, and newsletters.

### 2.4. Study Selection

Articles were imported into EndNote (version x9, Clarivate Analytics. Philadelphia PA) and duplicates removed using the “find duplicates” function and review by authors. Articles were imported into Rayyan (a collaboration platform designed for systematic reviews [26]) for screening and further de-duplication.

An abstract review form was developed, based on the selection criteria and piloted with 40 abstracts by five reviewers (S.H., R.S., J.K., O.L., and A.M.B.). Two reviewers (T.N. and J.K.) independently screened 30% of the titles and abstracts. Irreconcilable differences were resolved by a third reviewer (A.M.B). One reviewer (T.N.) screened the remaining titles and abstracts and a second reviewer (A.M.B) reviewed those excluded. One reviewer (T.N.) screened all full-text articles and a second reviewer (A.M.B) reviewed excluded articles.

### 2.5. Data Extraction

A data extraction form was adapted from the Cochrane data extraction template, and two reviewers (T.N. and A.M.B.) independently pilot-tested it on three studies. The final form included the following fields: reference, country, participants (number, characteristics, diagnoses), study design, level of evidence (based on the AACPDM classification [27]), setting, intervention, comparison, outcomes (measures and time points), aspects of HL assessed (access, understanding, appraisal, use of information), result for each outcome, adverse events, and comments. One reviewer (T.N.) extracted data for the remaining studies and a second (A.M.B.) checked data for correctness and completeness. Discrepancies were resolved through discussion. The corresponding author of one study was asked for further information, but none was received.

### 2.6. Risk of Bias (Quality) Assessment

One reviewer (T.N.) assessed each paper using the quantitative randomized controlled and non-randomized scales of the Mixed Methods Appraisal Tool (MMAT) Version 2018 [28], with full verification by a second reviewer (A.M.B.), any differences being resolved by discussion. Each scale has seven quality assessment criteria assessed using three response options (yes, no, and cannot tell).

### 2.7. Data Synthesis

Findings from included papers were synthesized in tables and descriptive narrative summaries. No meta-analysis was carried out, because studies had different interventions and outcome measures. HL outcomes were classified (following the definition [11]) as accessing, understanding, appraising, and/or applying health information by two raters (T.N. and A.M.B). Recommendations for practice were based on the grading of recommendations, assessment, development, and evaluation (GRADE) system [29] and the evidence alert traffic light system [30]. In accordance with the evidence-informed recommendations for rapid reviews [21], two stakeholders (parents of children with neurodevelopmental conditions) were included in the team (SH, RS).

## 3. Results

### 3.1. Study Selection

**Figure 1:**
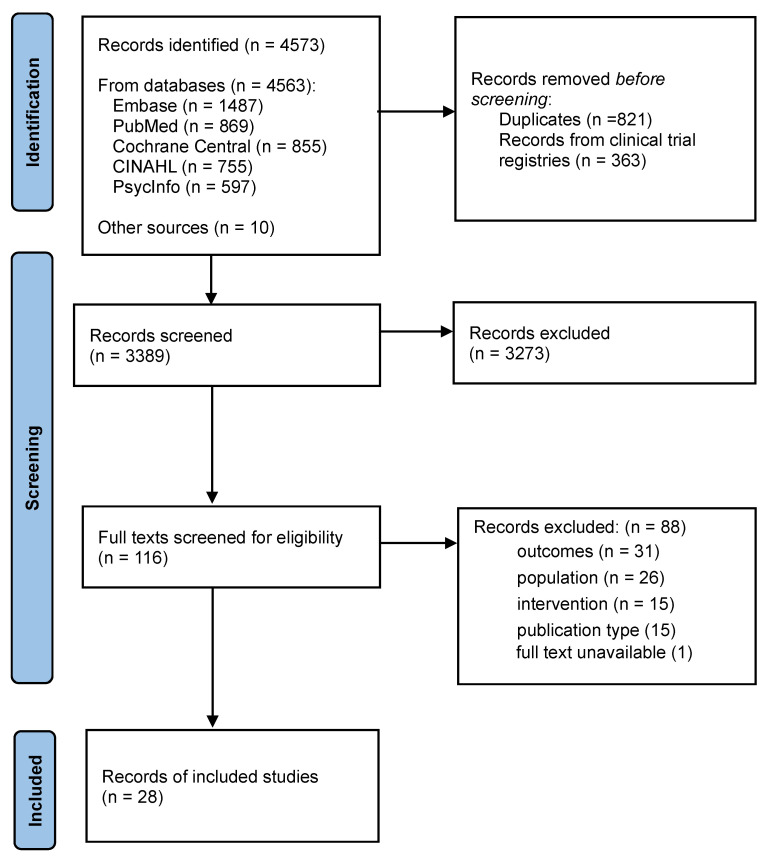
Preferred reporting items for systematic reviews and meta-analyses (PRISMA) flow diagram of literature searches and study selection.

There were 3389 unique records, of which 116 full texts were assessed for eligibility (Figure 1). Included in the review were 28 papers, reporting 26 studies (2 studies appearing in 2 papers each [31,32,33,34]).

### 3.2. Study Characteristics

Of the 26 studies, 11 were from the USA, 2 each from Australia, China, the Netherlands, and Spain, and 1 each from Aotearoa New Zealand, Canada, Germany, Iran, Italy, Türkiye, and the UK (Table 1). There were 13 randomized controlled trials (8 at Level I [33,34,35,36,37,38,39,40,41], 5 at Level II [31,32,42,43,44,45]), 2 non-randomized controlled trials (Level III) [46,47,48], and 11 Level IV studies (10 one-group pretest–post-test designs [49,50,51,52,53,54,55,56,57,58] and one case study [59]).

A total of 2979 participants commenced and 2232 completed the study interventions and assessments (ranging across studies: 3–306 who commenced, 3–253 who completed). Four studies targeted caregivers of adults (aged > 21 years) [37,38,49,51]; the remainder (*n* = 22) targeted caregivers of children and adolescents. Two studies targeted caregivers with only primary or secondary school education [38,42]; in three studies, most caregivers had tertiary education [54,57,58]; and eight studies included caregivers with education levels ranging from primary school to university [35,36,37,40,41,43,50,56]. The remaining 13 studies did not describe caregivers’ education levels.

In five studies, individuals had neurodevelopmental conditions: attention deficit hyperactivity disorder [56,57], Duchenne muscular dystrophy [59], moderate to severe disability (including autism spectrum disorder, developmental delay, and cerebral palsy) [41], and developmental concerns [42]. In 18 studies, they had chronic conditions: asthma [31,32,33,34,40,43], food allergies [52,55,58], eczema [35,44], cancers [37,49], epilepsy [45], hypertension [38], kidney disease [36], mood and anxiety disorders [48], obesity [39], oesophageal atresia [46,47], and type I diabetes [53]. The remaining five studies had mixed samples, probably including some with neurodevelopmental conditions: children needing nutritional support [50], adults with complex care needs [51], and children with nonspecific medical conditions or disabilities [54]. As it was impossible to separate individuals with neurodevelopmental conditions from those with chronic health conditions, they were pooled. Only three studies [42,48,50] targeted caregivers of recently diagnosed individuals; the other studies targeted caregivers of individuals, all or most of whom had been diagnosed months or years earlier.

The interventions used various media: online (*n* = 9) [35,36,45,46,47,49,52,53,56,57], group sessions (*n* = 8) [33,34,38,39,46,47,48,54,55,58], written materials (*n* = 6) [31,32,33,34,40,41,50,51], one-to-one sessions (*n* = 4) [31,32,35,51,59], video (*n* = 2) [42,43], phone calls (*n* = 2) [37,51], and text messages (*n* = 2) [42,44]. Five studies used multiple media [31,32,35,42,46,47,51], and one offered the intervention in two alternative media [33,34]. Caregivers helped develop at least eight of the interventions [40,42,49,52,53,54,55,57].

In the 15 studies with a control group, the comparison was no intervention or usual care (*n* = 8) [31,32,35,36,41,42,44,45,46,47] or an alternative intervention (*n* = 7) [33,34,37,38,39,40,43,48].

**Table 1:** Study characteristics.

Study, Country	Level of Evidence, Research Design	Participant and Child Characteristics and *n* Who Completed	Medium of Intervention	Intervention	Comparison
**Randomized controlled trials (Levels I-II)**					
Horner 2004, 2006 [31,32] USA	II Cluster RCT 3	60 commenced; 44 completed. Parent–child (7–11 y) dyads with **asthma**.	Booklet + in-person home visits	9 classes for chn over 1 m + booklet for parents on asthma mgt, pathophysiology, meds, qs for physician, and asthma mgt plan + home visits.	No intervention
Macy et al., 2011 [43] USA	II RCT	126 commenced; 86 completed (I gp: *n* = 42; C gp: *n* = 44). Parents of chn (4–12 y) presenting to ED with prior **asthma** Dx or Hx of wheeze. Educ level: <2° school to college.	Video	20 min video shown to parents in ED, incl asthma facts, meds, and pt skills.	Written educ materials with same content as video
Kintner et al., 2015a 2015b [33,34] USA	I RCT	205 caregiver–child (9–12 y) dyads (I gp: *n* = 117; C gp: *n* = 88) with **asthma** commenced; 136 completed all Ax at 24 m f-u.	In-person gp presentation or booklet	10 × 50 min asthma educ lessons for chn on asthma + for caregivers either 1 × 90 min info-sharing session or booklet.	6 × 50 min lessons for chn + bullet-pointed handouts for caregivers
Yin et al., 2017 [40] USA	I RCT	217 parents of chn (2-12 y) with **asthma** (I gp: *n* = 109, C gp: *n* = 108). Educ level: < high school grad (24%) to ≥college (59%).	Action plan	Low-literacy, plain language, pictogram-, and photograph-based asthma action plan.	Standard action plan
Jimenez et al., 2017 [42] USA	II RCT	64 parent–child (>3 y) dyads, (I gp: *n* = 31; C gp: *n* = 33) referred to EI for **dev concerns**. Max parent educ level: high school (85%).	Video + SMS	3 min video on child dev and EI pgm + 1 SMS sent 7–14 days later.	Standard care + publicly available handout on EI
Singer et al., 2018 [44] USA	II RCT	41 commenced; 30 completed. Parents of chn (0–3 y) with **eczema** (I gp: *n* = 14, C gp: *n* = 16).	SMS	Text messages with info on eczema sent daily for 42 d or until f-u appointment.	Usual care
Geense et al., 2018 [36] Netherlands	I RCT	146 commenced; 51 completed. Parents of chn with **chronic kidney disease** (I gp: *n* = 68; C gp: *n* = 65); 50% with high educ level.	Online	6 m access to 1) website with info/videos about kidney diseases, treatment, diet, and finance + chat room to message other parents and health care professionals; 2) web-based training platform (4 modules).	Usual care
Heckel et al., 2018 [37] Australia	I RCT	216 cancer pt–caregiver dyads commenced (*n*=108 per gp); 158 completed (I gp: *n* = 81; C gp: *n* = 77). Pts’ mean age 59 y; 88% **solid cancers**; receiving chemo (38%), radiotherapy (33%), or combination (29%); caregivers mean age 56 y; usually spouse (79%); educ level: 1° school to uni.	Phone	3 phone calls over 4 months from specialist oncology nurses, to discuss: psychological distress, health literacy, physical health, family support, financial burden, and practical difficulties.	3 phone calls from research personnel, supplying info line number to self-initiate contact.
Zhou et al., 2020 [41] China	I RCT	306 parent–child (2–6 y) dyads (I gp, *n* = 156, C gp, *n* = 150) with **mod–severe disability (ASD, dev delay, CP)**. Educ levels: <9 y (14%) to ≥14 y (57%). N = 253 completed, but ITT analysis on *n* = 306.	Educ materials	Structured package of oral health educ materials on brushing, tooth-friendly eating, and dental visits for parents + social stories for chn.	Standard leaflets issued by the Health Department.
Tutar Güven et al., 2020 [41] Türkiye	II RCT	70 child–parent dyads commenced; 69 completed (I gp: *n* = 35; C gp: *n* = 34). Chn (9–18 y) with **epilepsy** and no disability or other chronic disease.	Online	12 w access to web-based epilepsy educ pgm with info about epilepsy, Tx, and 1st aid.	Usual care
Cheng et al., 2021 [35] China	I RCT	136 parent–child dyads (*n* = 68 per gp). Chn (3–12 y) with mod or severe **eczema**. 133 completed. ITT analysis. Educ level: 56% post-secondary.	1-to-1 in-person educ session + online group	Parental eczema educ incl 30 min nurse-delivered sessions + 3 m online group sharing + standard eczema treatments.	Standard eczema treatments
Noroozi et al., 2022 [38] Iran	I Cluster RCT	200 pts (M = 61 y; 77% F; 77% homemakers) with **hypertension** + mother (food preparers) of family (100 per gp). Pt educ level: 95% 1° school. (N of mothers not specified; unclear how many pts were also mothers.)	In-person gp	2 d info workshop on hypertension and diet + ongoing routine educ from healthcare wkrs.	2 d info workshop on parenting styles + ongoing routine educ from healthcare wkrs.
Te’o et al., 2022 [39] Aotearoa New Zealand	I RCT	Parent–child (4–16 y) dyads with **overweight/obese** chn. 161 commenced; 145 at 12 m; 120 at 24 m; 80 at 60 m.	In-person family gp sessions	12-month multidisciplinary pgm of weekly physical activity or nutrition sessions + 6-monthly home-based Ax and advice.	6-monthly home-based Ax and advice.
**Non-randomized controlled trials (Level III)**					
Sapru et al., 2016 [48] Canada	III Non-RCT	19 commenced; 16 completed. Caregivers of chn (6–12 y, M = 8 y) referred with **mood and anxiety disorders** (in-person: *n* = 10; online gp: *n* = 6).	In-person gp sessions	3 × PPT presentations with same content as in-person sessions, emailed to family over 3 w	3 × 1 h in-person gp sessions over 3w about Tx options, interpersonal and communication skills, and problem-solving and reflect + gp social interactions
Dingemann et al., 2017 [46,47] Germany	III Non-RCT	29 adolescent pts (14–21 y) with **oesophageal atresia** (I gp: *n* = 10; C gp: *n* = 19) + 25 parents (I gp: *n* = 7; C gp: *n* = 18). F-u data for 22–23 adolescents and 9–23 parents (*n* varying across subscales).	In-person group workshops + online support.	2 d pgm incl 12 × 45 min modules with info on changes on turning 18; changing doctor; healthcare system; career; social networks; medical issues; coping + parent-specific component: social legislation + medical and psychological issues.	Usual care
**One-group and case series designs (Level IV)**					
LeBovidge et al., 2008 [58] USA	IV One-group pretest–post-test	59 parents of 61 chn (5–7 y) with **food allergies** commenced. Educ level: 87% college or graduate degree. 48 completed f-u.	In-person group	3.5 h workshop to promote parents’ coping and reduce stress and burdens in food allergy mgt.	None
Arikian et al., 2010 [59] USA	IV Case series	Families of 3 **non-ambulatory obese boys with Duchenne muscular dystrophy**, aged 8, 13, 13 y.	1-to-1 in-person meetings	20 × sessions and written materials on childhood obesity, weight loss, and prevention, over 6 months.	None
Ossebaard et al., 2010 [57] Netherlands	IV One-group pretest–post-test	12 parents of chn (M = 8 y) with **ADHD** (0.2% of 7500 who visited site and completed q’aires before and after using site). Educ level: most > average.	Online	Web-based decision aid on ADHD	None
Ryan et al., 2015 [56] UK	IV One-group pretest–post-test	91 caregivers of chn (4–18 y, M = 10 y) with **ADHD or suspected ADHD** (172 commenced and 158 completed, but only 91 accessed website). Caregiver educ levels: <2° school to postgraduate.	Online	Info-based website on ADHD mgt	None
Contreras-Porta et al., 2016 [55] Spain	IV One-group pretest–post-test	184 commenced; 174 completed. Parents of chn (M = 4.9 y) with **food allergy**.	In-person group workshops	2 × 2 h workshops on food allergies delivered by physicians and expert parents, incl 7 educ videos. (In-person version of intervention reported below [52].)	None
Armstrong-Heimsoth et al., 2017 [54] USA	IV One-group pretest–post-test	30 mothers and staff-parent advocates of individuals with **nonspecific medical conditions or disabilities**. Complete data from 24–28 (*n* varying across subscales). Educ level: some college.	In-person group workshop	1 × 60 min session about how and where to look for reliable health information online, how to form a searchable question, how to share their findings with their healthcare providers, and how to use information delivery shortcuts such as email alerts.	None
Holtz et al., 2018 [53] USA	IV One-group pretest–post-test	50 commenced; 46 completed. Parents of chn (5–18 y) with **T1D**.	Online	8 w access to website with info on navigating life events, dev milestones with T1D, links to websites/resources + closed Facebook parent gp.	None
Ruiz-Baqués et al., 2018 [52] Spain	IV One-group pretest–post-test	207 commenced; 130 completed. Caregivers of chn (M = 5 y) with **food allergies**.	Online	2 w access to a 5 h online educ pgm on food allergy mgt. (Online version of intervention reported above [55].)	None
Guida et al., 2019 [51] Italy	IV One-group pretest–post-test	47 caregivers (sons, spouses, siblings, parents, 83% F, aged 30 to 80) of adults with **complex care needs**.	1-to-1 in-person + phone + booklet	2 motivational sessions in 1 m + handbook incl managing info and communication, navigating healthcare settings and personal wellbeing.	None
Buchhorn-White et al., 2020 [50] Australia	IV One-group post-test only	30 commenced, 18 completed. Parents of chn **needing nutritional support** (most [78%] enteral nutrition or NG tube) during hospital stay (excl ICU). Educ level: Y10 to university degree.	Decision aid booklet	During their children’s hospital stay, parents read decision aid booklet on the risks and benefits of oral nutrition support, nasogastric and gastrostomy tube feeding and total parenteral nutrition.	None
Merz et al., 2022 [49] USA	IV One-group pretest–post-test for caregivers; (II RCT for pts)	10 caregivers + 50 pts commenced; 8 caregivers + 45 pts completed. Pts (50–64 y) with **solid cancers**, stages 3 and 4. Pts randomized to I and C gps, but all caregivers in I gp. (Only caregivers are considered in this review.)	Mobile app	12 w access to app with supportive care services and resource info	None for caregivers

Abbreviations: 1°, primary; 2°, secondary; ADHD, attention deficit hyperactivity disorder; ASD, autism spectrum disorder; Ax, assessment; C gp, control group; chn, children; CP, cerebral palsy; d, day; dev, developmental; Dx, diagnosis; ED, emergency department; educ, education; EI, early intervention; excl, excluding; F, female; f-u, follow-up; gp, group; h, hour; Hx, history; I gp, intervention group; incl, including; info, information; ITT, intention to treat; M, mean; max, maximum; meds, medications; mgt, management; min, minutes; m, month; mod, moderate; *n*, number; pgm, program; pt, patient; q’aire, questionnaire; qs, questions; RCT, randomized controlled trial; T1D, Type 1 diabetes; Tx, treatment; w, week; wkrs, workers; y, years.

### 3.3. Risk of Bias Within Studies

Using the MMAT for quantitative randomized controlled trials (Level I–II studies), one study [31,32] did not meet the screening criteria (due to insufficient reporting of inclusion criteria, intervention, group sizes, standard deviations, and significance levels). The remaining 12 studies ranged in quality from 20% to 100% (Table 2). Nearly all these 12 studies’ groups were comparable at baseline (*n* = 11), and participants adhered to the intervention (*n* = 10). About half performed randomization appropriately (*n* = 6) and had complete outcome data (*n* = 7) and blinded assessors (*n* = 6).

Using the MMAT for quantitative non-randomized trials (Level III–IV studies), one study [57] did not meet the screening criteria (due to lack of an explicit research question and lack of data from 99% people who visited the website). The remaining 12 studies ranged in quality from 20% to 80%. Most of these 12 studies had complete outcome data (*n* = 9) and administered the intervention as intended (*n* = 10), with half using appropriate measures (*n* = 6); however, confounders were generally not accounted for (*n* = 1), and in none of the studies were participants shown to be representative of the population (*n* = 0).

### 3.4. Results of Individual Studies

Table 3 shows the studies’ outcomes. Caregiver-related outcomes included knowledge [38,39,40,42,43,44,45,46,47,48,49,52,53,55,56,57] (*n* = 15), management of the condition [31,32,33,34,35,36,38,40,41,45,51,57,59] (*n* = 11), self-efficacy or self-perceived understanding, competence, or control [35,36,37,43,50,51,53,54,58] (*n* = 9), HL [37,45,52] (*n* = 3), use of services [42,49] (*n* = 2), attitudes [38,42] (*n* = 2), and communication with healthcare professionals [36,52] (*n* = 2). Secondary outcomes related to the individual with the health condition; these were health indicators [35,38,41,43,44,59] (*n* = 6) and the quality of life or health-related quality of life of the person with a chronic condition [36,46,47,59] (*n* = 3). HL outcomes were classified, according to the definition of HL [11], into accessing (*n* =7), understanding (*n* = 22), appraising (*n* = 2), or using (*n* = 18) information.

Seven of the 13 randomized controlled trials reported significant between-group effects. The conclusions from these trials are summarized in Table 4. Of the remaining six randomized controlled trials, two studies reported between-group differences on only two items in purpose-designed questionnaires, with no significant effects for any other items [33,34,42]. No other trials reported significant between-group effects.

Two non-randomized controlled trials reported no significant group differences [46,47,48].

Of the 13 evidence level IV studies, 7 one-group pretest–post-test designs reported significant improvements in caregiver knowledge and self-efficacy after intervention, but the quality of evidence was low (Evidence level IV, 20–40% on MMAT) [50,52,53,54,55,56,58]. A level IV case series with three children (60% on MMAT) also reported improvements in caregiver management and child quality of life following intervention [59].

### 3.5. Grading of Evidence and Recommendations

Using the GRADE system for rating strength of evidence [29], 11 studies were graded as high [33,34,35,36,37,38,39,40,41,42,44,45], 3 as moderate [43,46,47,48], 9 as low [31,32,49,51,53,54,55,56,58,59], and 3 as very low [50,52,57]. No studies reported adverse effects from interventions, though only one study mentioned monitoring for them [33,34]. Recommendations were “probably do it” for the six interventions arising from studies with moderate and high strength of evidence for significant between-group effects (online Supplemental Table S2).

Using the traffic light rating system [30], there was insufficient evidence to recommend any of the interventions unreservedly (“Green: go”), as there were not multiple controlled studies supporting any intervention; nor were there reasons (e.g., adverse effects) to recommend avoiding any interventions (“Red: stop”). All interventions were rated as “Yellow: measure”, meaning that the effect is uncertain, and outcomes should be measured when interventions are used.

## 4. Discussion

### 4.1. Interpretation of Findings

This rapid review shows that HL interventions for caregivers of individuals with neurodevelopmental and chronic conditions can improve caregivers’ HL and the individuals’ health-related outcomes across diverse conditions, ages, and carer education levels and using diverse intervention media (Table 4).

The studies included over 2000 individuals of all ages from 12 countries with over 17 different health conditions. Only a third of studies stated that they consulted target populations when developing the intervention, so future HL interventions may be strengthened through co-design with target populations [60].

The HL interventions used seven media (e.g., online, workshop, and print) and ranged in length from a single contact to a 12-month program. The interventions with moderate-to-strong evidence of effectiveness appeared to be more intensive than the ineffective interventions. Effective interventions lasted for 6 weeks, 3 months, and 12 months; one was a 2-day workshop with follow-up, and two used written materials. On the other hand, ineffective interventions from studies with moderate-to-strong evidence consisted of single videos, one 90 min group session, and three phone calls over 4 months. Only one unsuccessful intervention was intensive (6-month access to website), but that study had a high (65%) attrition rate [36]. Longer, intensive HL programs may, therefore, be more successful, but attention must be paid participant retention.

As Yuen et al. [20] observed, some aspects of HL are targeted more than others; most studies assessed caregiver *understanding* of the health condition (knowledge) and *application* of that knowledge (management of the condition). Very few studies assessed caregivers’ *access* or *appraisal* of health information; although, these are also key components of HL. Appraisal may involve advanced skills; one study that targeted appraisal used a university health sciences librarian to deliver training to caregivers with college education [54]. Nevertheless, this review found that at least some HL outcomes can be achieved with caregivers from all educational levels, including primary school only [38]. Service utilization, which has had mixed results in previous studies [14,60], was not enhanced by HL interventions in the only two studies that investigated it [42,49].

The strength of evidence across studies ranged from high to very low. The evidence for each of the effective interventions came from only one study of moderate-to-high strength of evidence, and so only weak recommendations could be made; traffic light ratings were yellow for all studies. Nevertheless, there is enough moderate to high level evidence to make six “probably do it” recommendations (online Supplemental Table S2). Even interventions without this level of evidence could be used in practice (as there were no reported adverse effects) if the outcomes are monitored.

### 4.2. Strengths and Limitations

This was a rapid review, so some steps of a systematic review were curtailed, following the evidence-informed recommendations for rapid reviews [21]. These included avoiding grey literature, limiting articles to English, and having most screening and all data extraction, risk of bias, and strength of evidence assessments carried out by a single author and verified by a second. There was no information specialist. The studies in this area are sparse and published across a range of disciplines, and so wide-ranging search terms had to be used to capture relevant papers. However, this meant that only 28 out of 3389 papers met the inclusion criteria, so there may be some subjectivity in their selection. Caregiver outcomes important for a holistic view of caregivers’ roles (e.g., advocacy skills) were not considered, nor were interventions that may have influenced caregiver HL without targeting it. Feasibility and acceptability of the interventions were not reported in this review; usability was assessed in only five studies [36,40,45,50,54], where it was found adequate or better.

### 4.3. Implications for Policy, Practice, and Research

HL interventions for caregivers of children and adults with neurodevelopmental and chronic conditions can improve caregiver HL and health outcomes. Intensive interventions (e.g., ≥3 months) may be required, but attention must be paid to retaining participants and delivering training in a digestible form. More attention should be given to caregivers’ access and appraisal of information. As HL intervention research is still sparse [20], there is abundant scope for research developing and assessing HL interventions, especially for caregivers of individuals with neurodevelopmental conditions.

## 5. Conclusions

Caregivers of individuals with neurodevelopmental and chronic health conditions have long-term daily responsibilities in managing health conditions, for which they require HL skills. This review found that HL interventions for caregivers of these individuals can sometimes improve caregiver HL and individuals’ health outcomes, but not always. Effective interventions appear to be more intensive but are otherwise diverse in their target populations and types of interventions. Future research is required to develop and assess HL interventions for these and similar populations.

## Figures and Tables

**Table 2:** Quality of studies according to the Mixed Methods Appraisal Tool (MMAT).

		Screening		Methodological Quality Criteria					Score/5 (%)
Study	Study Design	S1	S2	2.1	2.2	2.3	2.4	2.5	
Randomized controlled trials (Levels I–II)									
Horner 2004 2006 [31,32]	II Cluster RCT	Y	N	N	Y	N	?	?	1 (20%)
Macy et al., 2011 [43]	II RCT	Y	Y	?	?	N	Y	Y	2 (40%)
Kintner et al., 2015a 2015b [33,34]	I RCT	Y	Y	?	Y	N	Y	N	2 (40%)
Yin et al., 2017 [40]	I RCT	Y	Y	Y	Y	Y	Y	Y	5 (100%)
Jimenez et al., 2017 [42]	II RCT	Y	Y	Y	Y	Y	?	Y	4 (80%)
Singer et al., 2018 [44]	II RCT	Y	Y	Y	Y	N	Y	Y	4 (80%)
Geense et al., 2018 [36]	I RCT	Y	Y	Y	Y	N	?	Y	3 (60%)
Heckel et al., 2018 [37]	I RCT	Y	Y	?	Y	N	?	Y	2 (40%)
Zhou et al., 2020 [41]	I RCT	Y	Y	Y	Y	Y	Y	Y	5 (100%)
Tutar Güven et al., 2020 [45]	II RCT	Y	Y	?	Y	Y	?	Y	3 (60%)
Cheng et al., 2021 [35]	I RCT	Y	Y	Y	Y	Y	Y	Y	5 (100%)
Noroozi et al., 2022 [38]	I Cluster RCT	Y	Y	?	Y	Y	?	Y	3 (60%)
Te’o et al., 2022 [39]	I RCT	Y	Y	?	Y	Y	N	?	2 (40%)
**Study**	**Study design**	**S1**	**S2**	**3.1**	**3.2**	**3.3**	**3.4**	**3.5**	
Non-randomized controlled trials (Level III)									
Sapru et al., 2016 [48]	III Non-RCT	Y	Y	?	Y	Y	Y	Y	4 (80%)
Dingemann et al., 2017 [46,47]	III Non-RCT	Y	Y	?	Y/N	N	N	Y	2 (40%)
One-group and case series designs (Level IV)									
LeBovidge et al., 2008 [58]	IV One-group pretest–post-test	Y	Y	N	N	Y	N	Y	2 (40%)
Arikian et al., 2010 [59]	IV Case series	Y	Y	?	Y	Y	N	Y	3 (60%)
Ossebaard et al., 2010 [57]	IV One-group pretest–post-test	N	N	N	N	N	N	?	0 (0%)
Ryan et al., 2015 [56]	IV One-group pretest–post-test	Y	Y	?	Y	Y	N	N	2 (40%)
Contreras-Porta et al., 2016 [55]	IV One-group pretest–post-test	Y	Y	?	N	Y	N	Y	2 (40%)
Armstrong-Heimsoth et al., 2017 [54]	IV One-group pretest–post-test	Y	Y	N	N	Y	N	Y	2 (40%)
Holtz et al., 2018 [53]	IV One-group pretest–post-test	Y	Y	N	N	Y	N	Y	2 (40%)
Ruiz-Baqués et al., 2018 [52]	IV One-group pretest–post-test	Y	Y	?	N	N	N	Y	1 (20%)
Guida et al., 2019 [51]	IV One-group pretest–post-test	Y	Y	?	Y	Y	N	Y	3 (60%)
Buchhorn-White et al., 2020 [50]	IV One-group post-test only	Y	Y	N	N	N	N	Y	1 (20%)
Merz et al., 2022 [49]	IV One-group pretest–post-test for caregivers (II RCT for pts)	Y	Y	?	Y/N	Y	N	?	2 (40%)

Abbreviations: ?, unknown or uncertain; N, no; RCT, randomized controlled trial; Y, yes; Y/N, yes for at least one measure and no for at least one measure. S1, are there clear research questions?; S2 does the collected data allow us to address the research question? For Levels I–II designs: 2.1., is randomization appropriately performed?; 2.2. are the groups comparable at baseline?; 2.3. are there complete outcome data?; 2.4. are outcome assessors blinded to the intervention provided?; 2.5. did the participants adhere to the assigned intervention? For Levels III-V designs: 3.1., are the participants representative of the target population?; 3.2., are measurements appropriate regarding both the outcome and intervention (or exposure)?; 3.3., are there complete outcome data?; 3.4., are the confounders accounted for in the design and analysis?; 3.5., during the study period, is the intervention administered (or exposure occurred) as intended?

**Table 3:** Results of individual studies.

Study	Outcomes	Measures	Ax times	Aspects of HL Assessed	Results
Randomized controlled trials (Levels I–II)					
Horner 2004 2006 [31,32]	Parent and child asthma self-mgt	(1) Management Behaviour Survey (MBS) (2) Asthma Inventory for Children (AI-C).	Baseline, 6 and 12 m f-u	Understand, apply	NS diffs (*p* > 0.05) btn gps on either measure.
Macy et al., 2011 [43]	(1) Parental asthma knowledge (2) Parental sense of control in managing chn asthma (3) Morbidity	(1) Purpose-designed q’aire (2) Perceived Control of Asthma Q’aire (3) The Measures of Morbidity Q’aire	Baseline, 4–6 w f-u	Understand, apply	Btn-gp diffs not reported. (1) Ptcpts with low HL improved (*p* < 0.001) regardless of intervention; ptcpts with high HL improved (sig not stated) with video only. (2) No change (sig not stated) from pre–post for either gp. (3) Not reported.
Kintner et al., 2015a 2015b [33,34]	Asthma mgt behaviours	Asthma Health Behaviours Survey, a purpose-designed 34-item, 5-point scale q’aire incl episode mgt, risk prevention/reduction behaviours, and health promotion behaviours	Baseline, 1, 12, and 24 m f-u	Understand, apply	I gp had sig higher use of appropriate symptom mgt techniques (*p* = 0.039) and use of pillow protector (*p* < 0.001) than C gp. NS gp diffs (*p* > 0.05) for all other items.
Yin et al., 2017 [40]	Knowledge of which meds to use, spacer use, and appropriate emergency response	Interview qs requiring correct interpretation of action plan to obtain correct answers.	Once, after receiving action plan	Understand, apply	I gp made sig fewer errors than C gp in meds (*p* = 0.03), use of spacer (*p* < 0.001), but not seeking emergency help (*p* = 1.0).
Jimenez et al., 2017 [42]	(1) Knowledge and attitudes re dev delay and EI (2) EI intake and evaln	(1) Purposed-designed q’aire (14 qs on 6-point scale) (2) Parent report and chart review	(1) Pre- and post-test (2) 6 m f/u	Understand, apply	(1) I gp sig higher than C gp on 1/6 dev delay items (*p* = 0.02) and 1/8 EI items (*p* = 0.03); NS diff (*p* > 0.05) for all other items (2) NS gp diff on EI intake (*p* = 0.80) or evaln (*p* = 0.68)
Singer et al., 2018 [44]	(1) Knowledge about eczema (2) Eczema severity	(1) Purpose-designed 16-question multiple choice quiz (2) Eczema area severity index (EASI) score (dermatologist Ax)	8 w f/u	Understand, apply	(1) I gp sig higher than C gp (*p* = 0.04) (2) NS gp diff (*p* > 0.05)
Geense et al., 2018 [36]	(1) Self-efficacy in communicating with healthcare professionals (2) Family mgt of chronic conditions (3) Child’s QoL	(1) Perceived efficacy in patient–physician interactions (PEPPI-5) (2) Family management measure (FaMM) (3) Child vulnerability scale (CVS)	Baseline, 6 m f-u	Understand, apply	NS gp diffs (*p* > 0.05) in any outcomes
Heckel et al., 2018 [37]	(1) Caregiver health literacy (2) Caregiver empowerment	(1) Health literacy q’aire (HLQ) (2) Health educ impact q’aire (heiQ)	Baseline, 1 and 6 m f-u	Access, understand	NS gp diffs (*p* > 0.05) in HLQ and heiQ
Zhou et al., 2020 [41]	(1) Prevalence of caries (2) Oral hygiene and gingival status (3) Oral health behaviours	(1) dmft (decayed, missing, and filled primary teeth) index (2) Modified gingival index (MGI) and simplified debris index (DI-S) (3) Tooth brushing performance	Baseline, 24 m f-u	Apply	(1) NS gp diff (*p* > 0.05) (2) I gp had lower MGI and DI-S than C gp (*p* < 0.001). (3) I gp used tooth brushing steps and took longer (*p* < 0.01) than C gp.
Tutar Güven et al., 2020 [45]	(1) Parent and child knowledge of epilepsy (2) Parent and child e-health literacy (3) Parent epilepsy self-mgt skills (4) Child’s seizure self-efficacy	(1) Epilepsy knowledge test (EKT) (2) E-health literacy scale (eHEALS) (3) Pediatric epilepsy medication self-management q’aire (PEMSQ) (4) Seizure self-efficacy scale for children (SSES-C)	Baseline, 12 w f-u	Access, understand	I gp improved more (*p* < 0.001) than C gp on all outcomes.
Cheng et al., 2021 [35]	(1) Parents’ self-efficacy in implementing eczema Tx (2) Tx adherence (3) Eczema symptoms (4) Family QoL	(1) Parental self-efficacy with eczema care index (C-PASECI) (2) Problematic experiences of therapy scale (C-PETS). (3) Scoring atopic dermatitis (SCORAD), skin hydration (SH) transepidermal water loss (TEWL) (4) Family dermatology life quality index (C-FDLQI)	Baseline, 3 m f-u	Apply	(1) I gp improved > C gp (*p* < 0.001). (2) I gp improved > C gp (*p* < 0.001). (3) I gp improved > C gp on SCORAD, SH (*p* < 0.001) and TEWL (*p* = 0.045) (4) I gp improved > C gp (*p* < 0.001).
Noroozi et al., 2022 [38]	(1) Perceived susceptibility, severity, benefits, barriers, and self-efficacy (2) Salt consumption (3) Blood pressure	(1) Purpose-designed q’aire based on health belief model (2) Urine sodium level, creatinine, salt consumption (3) Systolic and diastolic BP	Baseline, 8 w f-u	Understand, apply	(1) I gp sig > C gp at f-u on perceived susceptibility, severity, benefits, and self-efficacy (all *p* = 0.001) and perceived barriers (*p* = 0.007). (2) I gp (but not Cp gp) improved sig (*p* < 0.05). (3) I gp (but not Cp gp) improved sig (*p* < 0.05).
Te’o et al., 2022 [39]	(1) Food knowledge (2) Physical activity knowledge	(1 and 2) Purpose-designed q’aire (based on National Survey of Chn and Young People’s Physical Activity and Dietary Behaviours in NZ) (%correct/incorrect).	Baseline, 12, 24, and 60 m f-u	Understand	(1) NS gp diffs (*p* > 0.05) (2) NS diffs at 12 or 24 mo. I gp sig > C gp at 60 m f-u (*p* < 0.05)
Non-randomized controlled trials (Level III)					
Sapru et al., 2016 [48]	Knowledge of mood and anxiety disorders	Understanding mood and disorders q’aire (UMDQ) and understanding of anxiety disorders q’aire (UMAQ)	Baseline, after intrvntn	Understand	NS gp diffs
Dingemann et al., 2017 [46,47]	(1) Transition specific knowledge (2) Patient activation = commitment to healthcare (3) HRQoL	(1) Purpose-designed multi-choice q’aire (2) Patient activation measure-13D (3) DISABKIDS chronic generic measure-37	Baseline, 4 w f-u	Understand, apply	(1) NS gp diff (*p* = 0.34). (2) NS gp diff (*p* = 0.27). (3) NS gp diff (*p* = 0.19–0.97).
One-group and case series designs (Level IV)					
LeBovidge et al., 2008 [58]	Parent-perceived competence in managing food allergies.	(1) Purpose-designed family coping with food allergy q’aire (FCFAQ)	Baseline, immediate post-test, 4-8 w f-u	Understand, apply	Sig imprvmt from baseline to post-test (*p* < 0.001) and from baseline to f-u (*p* < 0.001).
Arikian et al., 2010 [59]	(1) Family food environment (2) Children’s quality of life (3) Child and parent weight	(1) Food shelf inventory (FSI) (2) Pediatric quality of life inventory (PedsQL) (3) Digital weighing scales	Baseline, after 6 m Tx	Apply	Improved low fat foods on FSI (*n* = 3), child weight (*n* = 1), parent weight (*n* = 3), QOL (*n* = 3).
Ossebaard et al., 2010 [57]	(1) Decision-making skills (2) Knowledge of ADHD and treatment options	(1) Purpose-designed q’aire with 5-point scale (2) Purpose-designed q’aire using a 10-point scale	Before and after using decision aid on website	Understand, apply	NS changes in any outcome
Ryan et al., 2015 [56]	ADHD knowledge	30-item true/false q’aire, adapted from ADHD knowledge and opinion scale (AKOS-R)	Baseline, 1 m f-u	Access, understand	Sig increase in knowledge (*p* < 0.001)
Contreras-Porta et al., 2016 [55]	Food allergy knowledge	Purpose-designed q’aire		Understand, apply	Sig increase in 73% items in knowledge q’aire
Armstrong-Heimsoth et al., 2017 [54]	Self-rated confidence in getting, understanding, judging, and sharing health information	Purpose-designed q’aire incl 5 self-report items on 4-point scale + open-ended questions	Before and after training session	Access, understand, appraise	Sig imprvmts in self-ratings of finding info quality, judging info trustworthiness, retrieving info using alerts (all *p* < 0.001), and understanding info (*p* = 0.006), but not sharing info (*p* = 1.0)
Holtz et al., 2018 [53]	(1) Diabetes knowledge (2) Diabetes self-efficacy	Purpose-designed q’aire incl Likert-scale and true/false items	Baseline, 8 w f-u	Access, understand	(1) Sig imprvmt in knowledge (*p* = 0.006,) but not self-efficacy (*p* = 0.06)
Ruiz-Baqués et al., 2018 [52]	Knowledge about food allergies (e.g., symptoms, Dx, and Tx)	40-item purpose-designed q’aire using 5-point scale	Before and after pgm	Understand	Sig imprvmt in 15/30 items (*p* < 0.05) (total not reported)
Guida et al., 2019 [51]	(1) Health literacy (2) Caregiver self-efficacy (3) Caregiver engagement in healthcare mgt (4) Communication with healthcare professionals	(1) Health literacy 3-item q’aire (HLQ). (2) Revised scale for caregiver self-efficacy (S-Effic). (3) Caregiving health engagement scale (CHE-S) (4) Healthcare communication q’aire (HCCQ).	Baseline, 1 m f-u	Access, understand, appraise, apply	NS imprvmt in any outcomes (*p* > 0.05)
Buchhorn-White et al., 2020 [50]	Self-perceived imprvmt in understanding of nutrition	Purpose-designed q’aire items (4-point scale: not at all, a little, somewhat, or quite a bit)	After reading decision aid	Understand, apply	Most reported their understanding of nutrition (13/18), risks of malnutrition (14/18), benefits of nutrition support (17/18), and potential complications from nutrition support (13/18) improved “somewhat” or “quite a bit”.
Merz et al., 2022 [49]	Support care awareness and utilization.	(1) Purpose-designed supportive care awareness and utilization q’aire (2) Caregiver oncology quality of life (CarGOQoL) q’aire	Baseline, 12 w f-u	Access	NS change in caregiver awareness (*p* = 0.27) and utilization (*p* = 0.70)

Abbreviations: ADHD, attention deficit hyperactivity disorder; Ax, assessment; btn, between; C gp, control group; chn, children; dev, developmental; diff, difference; EI, early intervention; evaln; evaluation; f-u, follow-up; gp, group; HRQoL, health-related quality of life; I gp, intervention group; imprvmt, improvement; incl, including; info, information; meds, medications; mgt, management; m, month; N, no; NS, no statistically significant; ptcpts, participants; pts, patients; q’aire, questionnaire; QoL, quality of life; qs, questions; re, regarding; sig, significance, significant, or significantly; w, week; Y, yes.

**Table 4:** HL interventions with moderate-to-high strength of evidence of between-group effects.

Study	Level of Evidence	MMAT Score	Result
Yin et al., 2017 [40]	I	100%	A pictorial action plan in simple English for caregivers of children with asthma improved caregiver knowledge more than a standard action plan.
Singer et al., 2018 [44]	II	80%	Daily text messages for 6 weeks to caregivers of children with eczema improved caregiver knowledge more than usual care.
Zhou et al., 2020 [37]	I	100%	Educational materials about oral health given to caregivers of children with moderate to severe disability improved dental management and child dental health indicators
Tutar Güven et al., 2020 [45]	II	60%	Twelve weeks’ access to an epilepsy website for caregivers of children with epilepsy improved caregiver knowledge, HL, and management of the condition more than usual care.
Cheng et al., 2021 [35]	I	100%	Eczema one-to-one education sessions plus 3-month online group sharing plus standard eczema treatments for caregivers of children with eczema improved child symptoms, caregiver self-efficacy, and management of the condition.
Noroozi et al., 2022 [38]	I	60%	A 2-day workshop on hypertension and diet plus ongoing routine education by healthcare workers to caregivers of adults with hypertension improved caregiver knowledge, attitudes, and management of the condition more than a 2-day workshop on parenting styles plus ongoing routine education by healthcare workers.
Te’o et al., 2022 [39]	I	40%	A 12-month multidisciplinary programme of weekly physical activity or nutrition sessions plus 6-monthly home-based assessment and advice to caregivers of obese children improved caregiver and child knowledge more than 6-monthly home-based assessment and advice.

## Data Availability

Data extracted from included studies and used for analyses are provided in this paper.

## Supplementary Materials

The following supporting information can be downloaded at: https://www.mdpi.com/article/10.3390/children12010009/s1, Table S1: Search Strategy; Table S2: Grading of evidence and recommendations using GRADE and the traffic light system.
