# Supplementary material for: Efficacy of Health Literacy Interventions for Caregivers of Individuals with Neurodevelopmental and Chronic Conditions: A Rapid Review
Source: Children (Basel). 2024 Dec 24;12(1):9. doi: 10.3390/children12010009 (PMC11764195; doi:10.3390/children12010009)
Supplement: Supplementary file 1 [file children-12-00009-s001.zip › Supplementary Table 2.docx]

**Table S2:**

**Grading of evidence and recommendations using GRADE and Traffic Light System**

| **Studies** | **MMAT (% of items scoring Yes)^1^** | **Strength of evidence grade** | **Significant between-group effects** | **Adverse effects^2^** | **Recommendations to “probably do it”** | **Traffic Light System^3^** |
| --- | --- | --- | --- | --- | --- | --- |
| **Randomized Controlled Trials (Levels I-II)** | | | | | | |
| Horner 2004 2006 [27, 28] | 20% | Low | No | ? |  | Yellow |
| Macy et al 2011 [39] | 40% | Moderate | No | ? |  | Yellow |
| Kintner et al 2015a 2015b [29, 30] | 40% | High | Not for majority of items | No |  | Yellow |
| Yin et al 2017 [36] | 100% | High | Yes | ? | Use a pictorial Action Plan in simple English (rather than a standard Action Plan) for caregivers of children with asthma to improve caregiver knowledge. | Yellow |
| Jimenez et al 2017 [38] | 80% | High | Not for majority of items | ? |  | Yellow |
| Singer et al 2018 [40] | 80% | High | Yes | ? | Send daily text messages to caregivers of children with eczema for 6 weeks to improve caregiver knowledge. | Yellow |
| Geense et al 2018 [32] | 60% | High | No | ? |  | Yellow |
| Heckel et al 2018 [33] | 40% | High | No | ? |  | Yellow |
| Zhou et al 2020 [37] | 100% | High | Yes | ? | Provide educational materials about oral health to caregivers of children with moderate to severe disability to improve dental management and child dental health indicators. | Yellow |
| Tutar Güven et al 2020 [41] | 60% | High | Yes | ? | Provide 12 weeks access to an epilepsy website to caregivers of children with epilepsy to improve caregiver knowledge, health literacy, and management of the condition. | Yellow |
| Cheng et al 2021 [31] | 100% | High | Yes | ? | Provide eczema one-to-one education sessions + 3-month online group sharing + standard eczema treatments to caregivers of children with eczema to improve caregiver self-efficacy and management of the condition, family quality of life, and child symptoms. | Yellow |
| Noroozi et al 2022 [34] | 60% | High | Yes | ? | Provide a 2-day workshop on hypertension and diet + ongoing routine education by healthcare workers to caregivers of adults with hypertension to improve caregiver knowledge, attitudes, and management of the condition. | Yellow |
| Te’o et al 2022 [35] | 40% | High | Yes | ? | Provide a 12-month multidisciplinary programme of weekly physical activity or nutrition sessions + 6-monthly home-based assessment and advice to caregivers of obese children to improve caregiver and child knowledge. | Yellow |
| **Non-randomized controlled trials (Level III)** | | | | | | |
| Sapru et al 2016 [44] | 80% | Moderate | No | ? |  | Yellow |
| Dingemann et al 2017 [42, 43] | 40% | Moderate | No | ? |  | Yellow |
| **One-group and case series designs (Level IV)** | | | | | | |
| LeBovidge et al 2008 [54] | 40% | Low |  | ? |  | Yellow |
| Arikian et al 2010 [55] | 60% | Low |  | ? |  | Yellow |
| Ossebaard et al 2010 [53] | 0% | Very low |  | ? |  | Yellow |
| Ryan et al 2015 [52] | 40% | Low |  | ? |  | Yellow |
| Contreras-Porta et al 2016 [51] | 40% | Low |  | ? |  | Yellow |
| Armstrong-Heimsoth et al 2017 [50] | 40% | Low |  | ? |  | Yellow |
| Holtz et al 2018 [49] | 40% | Low |  | ? |  | Yellow |
| Ruiz-Baqués et al 2018 [48] | 20% | Very low |  | ? |  | Yellow |
| Guida et al 2019 [47] | 60% | Low |  | ? |  | Yellow |
| Buchhorn-White et al 2020 [46] | 20% | Very low |  | ? |  | Yellow |
| Merz et al 2022 [45] | 40% | Low |  | ? |  | Yellow |

^1^ Following the scoring used by Yuen E, Wilson C, Adams J, Kangutkar T, Livingston PM, White VM, et al. Health literacy interventions for informal caregivers: systematic review. BMJ Support Palliat Care. 2024 Feb 7.

^2^ ? - cannot tell

^3^ Green - Go: Effective, therefore do it;
Yellow - Measure: Uncertain effect, therefore measure outcomes to determine if progress is made;
Red - Stop: Ineffective, therefore don’t do it.
