# Supplementary material for: Efficacy of Health Literacy Interventions for Caregivers of Individuals with Neurodevelopmental and Chronic Conditions: A Rapid Review
Source: Children (Basel). 2024 Dec 24;12(1):9. doi: 10.3390/children12010009 (PMC11764195; doi:10.3390/children12010009)
Supplement: Supplementary file 1 [file children-12-00009-s001.zip › Supplementary Table 1.docx]

**Table S1: Search strategy**

| “health literacy” OR “health literate” OR “health literacies” OR “health skill*” OR “health competenc*” OR “parent health education” OR “health understanding” OR “health promotion” OR “health aware*” OR “hygiene education” OR “Rapid Estimate of Adult Literacy in Medicine” OR “eHealth Literacy Scale” OR “Patient Activation Measure” OR “Digital Health Technology Literacy” OR “Health Education Impact Questionnaire” OR “Information and Support for Health Actions Questionnaire” OR “Newest Vital Sign” |
| --- |
| AND |
| child* OR pediatric* OR paediatric* OR adolescen* OR infan* OR "parent*" OR "mother*" OR "father*" OR "mum*" OR “mom” OR "dad*" OR "caregiver*” OR “guardian*” |
| AND |
| disabilit* OR disabled OR "intellectual disabilit*" OR "intellectually disabled" OR "learning disorder*" OR "learning disabilit*" OR "learning dysfunction*" OR "neurodevelopmental disorder*" OR "neurodevelopmental disabilit*" OR "developmental disorder*" OR "developmental disabilit*" OR "developmental delay*" OR retard* OR "mental* handicap*" OR epilep* OR seizure* OR convulsion* OR autis* OR ASD OR Asperger* OR ADHD OR "attention deficit" OR hyperactive* OR hyperkin* OR "minimal brain dysfunction*" OR "minimal brain damage" OR MBD OR "cerebral palsy" OR "spastic diplegi*" OR "little* disease" OR syndrome* OR "fetal alcohol" OR FASD* OR "tic disorder*" OR kernicterus OR "muscular dystroph*" OR deaf* OR blind* OR "hearing loss" OR "hearing impair*" OR "vision disorder*" OR "sensory impair*" OR "vision impair*" OR "low vision" OR "rare disease*" OR "rare diagnos*" OR "rare disabilit*" OR "orphan disease*" OR "orphan disabilit*" OR "orphan diagnos*" OR "motor skills disorder*" OR "developmental coordination disorder" OR "developmental co-ordination disorder" OR “dys-coordination” OR “minor neurological dysfunction" OR "motor delay disorder*" OR “perceptual-motor impairment*" OR "motor coordination difficult*" OR "motor learning difficult*" OR "mild motor problem*" OR "motor coordination problem*" OR "sensorimotor difficult*" OR "sensory integrative dysfunction" OR psychomotor OR "motor control and perception" OR apraxi* OR dyspraxi* OR "perceptual motor dysfunction" OR "minimal cerebral dysfunction" OR “chronic condition*” OR “long term condition*” OR “long-term condition*” OR “life*long condition*” OR “chronic health condition*” OR “long term health condition*” OR “long-term health condition*” OR “life*long health condition*” OR “chronic illness*” OR “long-term illness*” OR “long term illness*” OR “life*long illness*” OR “chronic disease” OR “long term disease*” OR “long-term disease*” OR “life*long disease*” OR “asthma*” OR “cystic fibrosis” OR diabet* OR obes* OR “mental disorder*” OR “mood disorder*” OR “psychological disorder*” OR “psychiatric disorder*” OR anxi* OR depress* OR “mental illness*” OR “obsessive compulsive disorder” OR OCD OR “chronic pain” OR arthriti* OR hypertension OR schizophrenia OR fibromyalgia OR osteoporosis OR “irritable bowel syndrome” OR IBS OR endometriosis OR eczema* OR “atopic dermatitis” OR cancer* OR anaemi* OR “otitis media” OR allerg* OR bronchitis OR stroke* OR “kidney disease*” OR “cardiovascular disease*” OR “heart failure” OR “vascular disease*” OR “chronic obstructive pulmonary disease” OR COPD |
| AND |
| intervent* OR program* OR support* OR learning* OR training OR strateg* |
